# Primary extramedullary plasmacytoma with diffuse lymph node involvement: a case report and review of the literature

**DOI:** 10.1186/s13256-019-2087-7

**Published:** 2019-05-22

**Authors:** Leonard Naymagon, Maher Abdul-Hay

**Affiliations:** 1 New York University School of Medicine and Mount Sinai School of Medicine, New York, USA; 2 New York University School of Medicine and New York University Perlmutter Cancer Center, 240 East 38th street, 19Floor, New York, NY 10016 USA

**Keywords:** Primary plasmacytoma, Plasma cell dyscrasia, Multiple myeloma, Lymph nodes, Bortezomib, Thalidomide

## Abstract

**Background:**

Primary plasmacytomas are localized proliferations of clonal plasma cells occurring in the absence of a systemic plasma cell dyscrasia such as multiple myeloma. Primary plasmacytomas most commonly manifest as solitary lesions of the bone or of the upper aerodigestive tract. Presentation in a lymph node is very uncommon and can often be initially mistaken for lymphoma. Because they are local phenomena, primary plasmacytomas are managed with local therapies such as radiation or, less commonly, excision. Multifocal presentations are rare and are often not amenable to local treatment modalities, thus requiring systemic therapies. Because of their rarity, standardized treatment guidelines are not established, and treatment paradigms borrow heavily from those employed in multiple myeloma. Multifocal presentation in lymph nodes is nearly unheard of with only seven such cases reported in the existing literature, only four of which were diffuse enough to require systemic therapy. Here we describe the most diffuse and widely distributed instance of primary lymph node plasmacytoma yet reported and present a description of its successful treatment with systemic therapy.

**Case presentation:**

A 71-year-old Asian man presented with progressive fatigue in the setting of diffuse hypermetabolic lymphadenopathy throughout his chest, abdomen, and pelvis. A diagnosis of lymphoma was initially suspected; however, a lymph node biopsy was consistent with plasmacytoma. A bone marrow biopsy was unremarkable, and no monoclonal protein was identified, establishing a diagnosis of primary extramedullary plasmacytomas of the lymph nodes. He was treated with a myeloma-like regimen consisting of four cycles of bortezomib/dexamethasone followed by two cycles of thalidomide/prednisone with improvement in symptoms and near complete resolution of prior hypermetabolic lymphadenopathy. He remains in remission over 18 months following completion of therapy.

**Conclusion:**

This case report and accompanying literature review highlight the exceedingly rare and easily misclassified entity of primary plasmacytoma of diffuse lymph nodes. Importantly, we demonstrate that this entity may be treated with, and demonstrate excellent response to, systemic therapies often employed in multiple myeloma.

## Introduction

Plasma cell neoplasms are characterized by the uncontrolled proliferation of malignant plasma cell clones. The most common plasma cell malignancy is multiple myeloma (MM), which is characterized by a significant burden (> 10%) of clonal plasma cells within the bone marrow, monoclonal protein in serum and/or urine, and systemic symptoms (most commonly hypercalcemia, renal insufficiency, anemia, and/or bone lesions) [1, 2]. Plasmacytomas are clonal proliferations of plasma cells that are cytologically and immunophenotypically indistinguishable from MM; however, they demonstrate strictly local growth, without bone marrow or broader systemic involvement [3]. The most common manifestation of primary plasmacytoma is as a single lesion of bone (solitary osseous plasmacytoma) [2]. Primary plasmacytomas not involving bone (extramedullary plasmacytomas) typically manifest as lesions of the upper aerodigestive tract [4, 5]. Primary lymph node plasmacytomas are exceedingly rare with fewer than 40 cases having been described in the literature to date [6, 7]. The vast majority of lymph node plasmacytomas are solitary lesions, with only seven documented cases involving multiple nodes.

Here we describe a highly atypical case of primary plasmacytoma with extensive lymph node involvement. This case demonstrates the most diffuse and widely distributed instance of primary plasmacytoma yet described, with enlarged hypermetabolic lymph nodes evident throughout the neck, chest, abdomen, and pelvis. In fact, the dramatic lymphadenopathy at presentation initially prompted suspicion for lymphoma, and the eventual diagnosis of a plasma cell neoplasm was quite a surprise. Due to its remarkable dissemination this case could not be treated with the usual local modalities (namely radiation therapy) employed in the overwhelming majority of patients with primary plasmacytoma. As such, systemic therapy had to be employed. As data for systemic treatment of primary plasmacytoma is minimal, we borrowed heavily from myeloma treatment paradigms. In fact, this is the first reported case describing the use of modern-style myeloma therapy for the treatment of diffuse primary plasmacytoma. The success of such therapy in this case demonstrates that diffuse primary plasmacytomas not amenable to local therapies may be treated safely and effectively with drugs commonly employed in MM, such as proteasome inhibitors and immunomodulatory agents.

## Case presentation

Our patient is a 71-year-old Asian man with a history of rheumatoid arthritis (RA), type II diabetes, chronic kidney disease (CKD), and Hodgkin’s lymphoma, which was stage IV, diagnosed 6 years prior to presentation, status post six cycles of adriamycin/bleomycin/vinblastine/dacarbazine (ABVD), with sustained complete remission; he presented with progressive fatigue and malaise of 2 months’ duration. He was a former tobacco smoker with a 30 pack year history; however, he had quit smoking 20 years before presentation. He had no history of alcohol or illicit substance use. He was retired from a previous career as a farmer. He had no contributory family history. His vital signs were within normal limits with temperature of 36.3 °C (97.3 °F), blood pressure of 128/78 mmHg, and a heart rate of 70 beats per minute. An examination was most notable for new cervical lymphadenopathy. Other examination findings included finger deformities attributed to his RA and normal neurological examination. A laboratory evaluation was notable for pancytopenia with leukocyte count of 2.6 × 10^9^/L, platelet count of 50 × 10^9^/L, and hemoglobin of 11 mg/dL. His chemistry studies including calcium, creatinine, and liver function test were within normal limits. Positron emission tomography (PET) computed tomography (CT) demonstrated multiple enlarged hypermetabolic lymph nodes throughout his neck, chest, abdomen, and pelvis, the largest measuring 2.2 cm × 1.2 cm. This prompted concern for recurrence of Hodgkin’s lymphoma or *de novo* development of non-Hodgkin’s lymphoma. A tissue biopsy of an enlarged hypermetabolic left inguinal lymph node was obtained, with pathology notable for sheets of CD138+, MUM1+, MYD88-, and lambda restricted plasma cells, consistent with a plasma cell neoplasm. A bone marrow biopsy performed shortly afterward demonstrated largely unremarkable trilineage hematopoiesis without increased plasma cells, and with no monoclonal plasma cells detected. Our patient demonstrated normal renal function and electrolytes. Serum albumin, serum protein, and serum protein gap were within normal limits. His plasma and urine were negative for monoclonal protein, with normal findings on serum protein electrophoresis (SPEP), urine protein electrophoresis (UPEP), serum immunofixation, serum free light chain assay, and urine free light chain assay. Based on the above data, a diagnosis of multiple diffuse extramedullary plasmacytomas of the lymph nodes was established. On diagnosis our patient was on metformin 1000 mg daily, Januvia (sitagliptin) 100 mg daily, glipizide 5 mg daily, Nexium (esomeprazole) 40 mg daily, Vesicare (solifenacin) 5 mg daily, folic acid 1 mg daily, nebivolol 10 mg daily, and tamsulosin 0.4 mg daily.

Treatment was promptly initiated with subcutaneously administered bortezomib 1.5 mg/m^2^ and orally administered dexamethasone 40 mg weekly for 3 weeks on and 1 week off. Our patient’s platelet count improved considerably following initiation of treatment, allowing for the addition of thalidomide. He received four cycles of bortezomib/dexamethasone followed by two continuous 28-day cycles of thalidomide 100 mg orally administered with prednisone. Thalidomide was chosen rather than lenalidomide due to our patient’s CKD and some persistent thrombocytopenia. His initial presenting symptoms improved throughout the course of therapy. An interval PET-CT was then done to assess response, and demonstrated near complete resolution of prior hypermetabolic lymphadenopathy with no new areas of fluorodeoxyglucose (FDG) avidity noted. He now remains in remission over 18 months following completion of therapy.

## Discussion

By definition, primary plasmacytomas demonstrate strictly localized growth and no systemic manifestations of plasma cell malignancy [8–10]. It is this anatomic localization which distinguishes primary plasmacytomas from MM, which by definition demonstrates broader systemic involvement (including bone marrow involvement and paraproteinemia) [11]. Because primary plasmacytomas are local phenomena, they may be treated and cured with local interventions such as involved field radiation. In contrast, because MM is a systemic disorder, it requires systemic treatments such as chemotherapy and/or immune therapy [11, 12]. Given these radical differences in treatment, it is of utmost importance to unequivocally distinguish between these two entities. In fact, a rigorous workup is required to definitively rule out systemic involvement and establish a diagnosis of primary plasmacytoma [13–15].

The remarkably diffuse anatomic dissemination noted in this case is highly atypical of primary plasmacytoma and the primary diagnostic challenge lay in excluding MM. In fact, primary plasmacytomas of multiple lymph nodes is such a rare entity (only seven cases in the existing literature, see Table 1), that a diagnosis of underlying MM seemed likely [6, 16–30]. Thus, great care and rigor had to be taken to definitely rule out underlying myeloma, and establish with certainty a diagnosis of diffuse lymph node plasmacytoma [31–33]; to do so required demonstration of an uninvolved bone marrow, absence of monoclonal immunoglobulins or light chains (both serum and urine), and absence of any of the clinical sequels of myeloma [34, 35]. It was only after frank myeloma had been thoroughly and exhaustively ruled out as above that our patient could be deemed to have diffuse primary plasmacytoma of lymph nodes [36].

**Table 1:** Reported cases of primary plasmacytoma with multiple lymph node involvement

Author/reference	Patient age/sex	Lymph nodes involved	Ig isotype	Serum monoclonal protein	Bone marrow involvement	Initial therapy	Response to initial therapy	Relapse or persistent disease	Additional therapy	Survival
Salem *et al*. [16]	48 M	Paratracheal, precarinal, subcarinal, aortopulmonary	IgG λ	M-Spike 2.1 g/dL, λ FLC 1613 mg/L	None	Involved field radiation (50 Gy in 25 fractions)	No response	Persistent disease following first-line radiation	VTD-PACE, then auto SCT, then lenalidomide maintenance	In CR 18 months following auto-SCT
Matsushima *et al*. [17]	56 F	Mandibular, cervical, axillary, para-aortic	IgA K	Present, though not quantified	None	Cyclophosphamide and prednisolone (10 courses)	Complete response (CR)	Relapse 6 years following initial CR	Melphalan and prednisolone (10C)	In CR 9 years following initial diagnosis
Gorodetskiy *et al*. [18]	48 F	Diffuse LAD, most prominent at supraclavicular, femoral nodes	IgA K	M-Spike 7.2 g/dL	None	CHOP for 9 cycles	Complete response (CR)	Relapse at 3 months and again at 19 months	Initial relapse treated with 1C CEVD, subsequent treated with 1C CHOEP	In CR 18 years following initial diagnosis
Menke *et al*. [6]	Not specified	“Disseminated LN involvement”, specific sites unspecified	Not specified	Not specified	None	Chlorambucil for 8 weeks	Complete response (CR)	None	None	In CR 3 years following initial diagnosis
Menke *et al*. [6]	Not specified	“Disseminated LN involvement”, specific sites unspecified	Not specified	Not specified	None	Chlorambucil and prednisone for 8 weeks	No response	Persistent disease following first-line therapy	None	Died of disease 2 months following diagnosis
Lim *et al*. [19]	56 M	Right submental and submandibular	IgG λ	M-Spike 1.6 g/dL	None	Involved field radiation (50 Gy in 25 fractions)	Incompletely reported	Residual mass of uncertain viability to be monitored with serial PET	None	Duration of survival not reported
Lin *et al*. [20]	58 F	Bilateral cervical	K light chain	Present, though not quantified	None	Excision	Complete response (CR)	None	None	In CR at 1 year following excision

Seven cases of primary plasmacytoma involving multiple lymph nodes are reported in the literature. Two of these cases (Lim *et al*. and Lin *et al.*) were localized enough to be treated via surgery or radiation alone. Thus, only five cases describe disease sufficiently disseminated as to require systemic chemotherapy

Abbreviations: *C* cycle, *CEVD* lomustine, etoposide, vinblastine, dexamethasone, *CHOP* doxorubicin, cyclophosphamide, vincristine, prednisolone, *CHOEP* doxorubicin, cyclophosphamide, vincristine, etoposide, prednisolone, *CR* complete remission, *F* female, *FLC* free light chain, *LAD* lymphadenopathy, *LN* lymph node, *M* male, *M-Spike* monoclonal spike, *PET* positron emission tomography, *SCT* stem cell transplantation,*VTD-PACE* dexamethasone, thalidomide, cisplatin, doxorubicin, cyclophosphamide, etoposide, bortezomib

Once the above diagnostic challenge was settled, there then emerged the therapeutic challenge. Solitary primary plasmacytoma is the only curable plasma cell disorder, and the treatment of choice is radiation therapy [14]. Treatment paradigms are less well established for those with multiple primary plasmacytomas. Certainly, local radiation may be an option for those with relatively few lesions where radiation fields and cumulative dosage would not be prohibitive. However, those with many diffuse lesions cannot feasibly be treated with purely local modalities. Such cases must then be treated with systemic chemotherapy. Given the rarity of multiple diffuse primary plasmacytomas, there is lack of disease-specific data, either prospective or retrospective, to guide therapy. As such, treatment paradigms must borrow heavily from those established for MM.

As the disease in this case was far too widespread to benefit from standard treatments for localized plasmacytoma (that is, radiation), our patient required systemic therapy. Due to the extreme paucity of data describing systemic therapy for primary plasmacytoma, treatment had to be planned largely without well-established precedent. Given the anatomically diffuse nature of the disease, it was deemed prudent to pursue a myeloma-like regimen. Bortezomib and dexamethasone were started initially. Thalidomide was initially withheld to avoid thrombocytopenia, however, it was added following platelet response to initial treatment (as above, thalidomide was chosen rather than lenalidomide due to our patient’s CKD and some persistent thrombocytopenia). Interval PET-CT after four cycles of bortezomib/dexamethasone followed by two cycles of thalidomide/prednisone demonstrated near complete resolution of prior hypermetabolic lymphadenopathy with no new areas of FDG avidity noted. Our patient remains in remission over 18 months following completion of therapy. Given this excellent response to initial therapy, and the absence of bone marrow involvement and monoclonal protein at diagnosis, his prognosis is felt to be relatively good, and the likelihood of progression to myeloma is felt to be relatively low [8–13].

## Conclusion

Solitary plasmacytomas, which typically manifest in bone or the upper aerodigestive tract and demonstrate no concurrent evidence of systemic myeloma, may be treated with purely local modalities such as involved field radiation. Multifocal plasmacytomas raise greater concern for concurrent occult myeloma; however, once systemic disease is ruled out, they too may be treated with local modalities if their extent and distribution are amenable. Multifocal primary plasmacytomas of lymph nodes are exceedingly rare and may be initially mistaken for lymphoma. Although systemic myeloma should be diligently ruled out, treatment may require the use of myeloma-like therapeutic regimens if local modalities are not anatomically feasible. Responses to myeloma-like regimens may be rapid, deep, and perhaps curative; however, a risk of future progression to systemic myeloma remains, particularly in cases with evidence of marrow involvement or monoclonal protein. It is not known why some plasma cell dyscrasias such as MM manifest systemically while others such as primary plasmacytomas manifest locally. A better understanding of the biologic mechanisms underlying these differences may potentially yield therapeutic insights for myeloma, as future treatments may aim to target those cellular elements most associated with diffuse or metastatic phenotypes.

## Abbreviations

ABVD: Adriamycin/bleomycin/vinblastine/dacarbazine
CKD: Chronic kidney disease
CT: Computed tomography
FDG: Fluorodeoxyglucose
MM: Multiple myeloma
PET: Positron emission tomography
RA: Rheumatoid arthritis
SPEP: Serum protein electrophoresis
UPEP: Urine protein electrophoresis
